# Loss aversion, the endowment effect, and gain-loss framing shape preferences for noninstrumental information

**DOI:** 10.1073/pnas.2202700119

**Published:** 2022-08-16

**Authors:** Yana Litovsky, George Loewenstein, Samantha Horn, Christopher Y. Olivola

**Affiliations:** ^a^Department of Banking and Finance, University of Innsbruck, Innsbruck, Austria, 6020;; ^b^Department of Social and Decision Sciences, Carnegie Mellon University, Pittsburgh, PA 15213;; ^c^Tepper School of Business, Carnegie Mellon University, Pittsburgh, PA 15213

**Keywords:** cognition, learning, judgment, decision-making, forgetting

## Abstract

We often talk about interacting with information as we would with a physical good (e.g., “consuming content”) and describe our attachment to personal beliefs in the same way as our attachment to personal belongings (e.g., “holding on to” or “letting go of” our beliefs). But do we in fact value information the way we do objects? The valuation of money and material goods has been extensively researched, but surprisingly few insights from this literature have been applied to the study of information valuation. This paper demonstrates that two fundamental features of how we value money and material goods embodied in Prospect Theory—loss aversion and different risk preferences for gains versus losses—also hold true for information, even when it has no material value. Study 1 establishes loss aversion for noninstrumental information by showing that people are less likely to choose a gamble when the same outcome is framed as a loss (rather than gain) of information. Study 2 shows that people exhibit the endowment effect for noninstrumental information, and so value information more, simply by virtue of “owning” it. Study 3 provides a conceptual replication of the classic “Asian Disease” gain-loss pattern of risk preferences, but with facts instead of human lives, thereby also documenting a gain-loss framing effect for noninstrumental information. These findings represent a critical step in building a theoretical analogy between information and objects, and provide a useful perspective on why we often resist changing (or losing) our beliefs.

## Significance

We build on Abelson and Prentice’s conjecture that beliefs are not merely valued as guides to interacting with the world, but as cherished possessions. Extending this idea to information, we show that three key phenomena which characterize the valuation of money and material goods—loss aversion, the endowment effect, and the gain-loss framing effect—also apply to noninstrumental information. We discuss, more generally, how the analogy between noninstrumental information and material goods can help make sense of the complex ways in which people deal with the huge expansion of available information in the digital age.

We live in an era defined by the availability of a seemingly limitless supply of information. Technology has enabled the creation of a vast, and exponentially growing, amount of data that may already exceed the combined storage capacity of all human brains (1, 2). It has also given us the ability to disseminate that information instantly and at negligible cost. These developments have yielded countless benefits but they have also created new problems (3), such as the spread of “fake news” (4) and dangerous conspiracy theories (5). These realities challenge not only the social good, but also the traditional perspective on how we acquire and evaluate information. According to conventional economics and decision theory, information is valued to the extent, and only to the extent, that it supports decisions which yield better outcomes (6). But this narrow view cannot explain many of the ways in which we actually engage with information, such as our willingness to pay for patently noninstrumental information (7) or our tendency to avoid important information that we think will adversely affect our beliefs (8–10). Although psychologists and economists have increasingly observed such complexities in people’s attitudes toward information (11, 12), we believe there is an important perspective missing from the conversation about how and why we value information that does not contribute to our welfare.

We follow up on Abelson (13) and Abelson and Prentice’s (14) insight that beliefs are treated much like physical possessions. Their key idea was that beliefs—which are often based on information that one thinks is true—are not merely valued as guides to interacting with the world, but as cherished possessions whose value could be disconnected from external outcomes. We propose that there is a similar relationship between the way people treat material objects and information. Just as research on the endowment effect—an asymmetry in preferences for acquiring versus giving up objects—helps explain why people are loath to give up their possessions, this perspective may illuminate why they often resist accepting that facts they think are true may be false. The notion that people grow attached to information just as they do physical goods was humorously suggested by Kahneman et al. (15), who joked that they were “naturally keener to retain” their belief in a scientific idea they had formed “than others might be to acquire it.”^*^ We corroborate their intuition by showing that two of the most important and extensively researched phenomena involving preferences for objects—loss aversion and diminishing sensitivity to both losses and gains from a reference point—also apply to information, even when that information is purely noninstrumental and thus cannot be translated into any material outcomes.

## Information Loss

Perhaps the most important general phenomenon regarding how people interact with material goods is loss aversion—the idea, from Prospect Theory (16), [see also (17)], that we feel worse about losses than we feel good about equivalent gains. Loss aversion accounts for various anomalous choice behaviors, such as the endowment effect (15), [although see (18, 19)]. Also integral to Prospect Theory is the notion of diminishing sensitivity to both losses and gains from a reference point (17, 20), which, when it comes to risk preferences, predicts a pattern of choices whereby people tend to be risk averse for gains and risk seeking for losses. Consequently, reframing a given set of outcomes as relative gains versus relative losses changes people’s risk preferences over that set. So far, loss aversion and diminishing sensitivity have been observed for various tangible outcomes, including nonconsumer outcomes such as human lives (21, 22), and a few nontangible resources, such as time (23–26), but not for information.

The current research is closest to a set of studies in information science conducted by Rafaeli and Raban (27, 28), who replicated the endowment effect for information in a game mimicking sales decisions. They gave participants the opportunity to buy and sell information, and observed greater willingness-to-accept prices than willingness-to-pay amounts. We extend their work in several critical ways. First, due to features of their elicitation method, Rafaeli and Raban attributed the endowment effect that they observed to risk aversion rather than loss aversion. Second, their observed preferences over information may be confounded by attitudes toward gaining and losing money, since their willingness-to-pay measure involved giving up money (which, due to loss aversion, people are reluctant to do), while their willingness-to-accept measure involved obtaining money. To avoid this issue, the studies reported herein do not involve money, but rather choices between different sets of information. Third, because the game they used was a novel task, there is significant uncertainty about the usefulness of the information involved. If that information was perceived to directly translate into financial rewards, it is impossible to determine whether the observed effect reflected loss aversion for information per se or for the in-game profits. To avoid this concern, our studies detach information from monetary and material gains by focusing on patently noninstrumental information in a familiar information environment. Moreover, we focus on noninstrumental information that is devoid of any belief-based utility (e.g., is not ego-relevant).

A few lines of research have directly considered or indirectly touched on the analogy between information and objects. For example, one theoretical account (29) proposes that information can be manifested in material ways (e.g., via tangible documents), but it does not make any claims about the way in which our valuations of information and objects are alike. Another account (30) models loss aversion over changes in beliefs and predicts that bad news about future consumption is more unpleasant than good news is pleasant. Among the scant empirical evidence is the recent finding that, as with physical objects, personally producing information increases its perceived value (31) [a phenomenon known as the “Ikea Effect” (32)]; in that study, however, the information—an online course about web accessibility—had potential instrumental value. The endowment effect has also been observed in the choices people make to exchange money for privacy (33) [see also (34)]. However, not wanting to give up personal information is inherently different from the desire to obtain (noninstrumental) information. In sum, existing research only provides suggestive evidence that preference patterns observed for goods may also apply to information. To the best of our knowledge, no prior work has provided empirical evidence of similarities between individuals’ preferences over valued goods and information that does not impact wealth or well-being.

Although loss aversion, the endowment effect, and gain-loss framing have been documented for various nonmaterial outcomes, such as jobs (35, 36), environmental public goods (37), and potential mates (38, 39), it is not clear^†^ that they would extend to information—especially noninstrumental information—which differs in many important respects from other material and nonmaterial outcomes examined in prior studies. First, the way we mentally represent losses of information may be very different from that of other resources for which loss aversion has been documented. For one, giving or receiving information does not come at the cost of the information itself. If we give someone money, objects, or time, we no longer have those resources to use for ourselves. By contrast, if we give someone information, we still retain that information. Losing information is also far harder to control. While it is possible to give up nearly any material possession, it is nearly impossible to voluntarily give up—i.e., to immediately forget—known information. This may explain why we avoid information that might negatively impact our beliefs (8–10). Perhaps an even more consequential difference is that information loss is more difficult to attend to and account for. Given the sheer quantity of information we consume, we cannot catalog everything we know as we could all the objects we own. As a result, it is harder to identify the loss of a piece of information from memory than the loss of a physical good: we generally do not realize that we have forgotten a piece of information until we try, and fail, to retrieve it from memory. This may contribute to the findings in the metacognition literature that we overestimate both our competence (47) and our knowledge (48).

Second, differences in the way people mentally represent losses of information versus objects may actually predict an *absence* of loss aversion for information. According to decision by sampling theory, loss aversion is not the product of a stable, internal system of valuation, but rather emerges from the way people compare the value of any given outcome to other values available in memory (22, 49). Given the distributions of financial losses and gains that people typically experience, larger amounts are more frequently observed for gains (e.g., monthly salary earnings) than for losses, whereas smaller amounts are more frequently observed for losses (e.g., daily purchases) than for gains. As a result, gains are more likely to be compared to larger amounts (previously observed gains), making them seem smaller, while losses are more likely to be compared to smaller amounts (previously observed losses), making them seem larger. With information, by contrast, it seems plausible that we are less aware of each discrete “loss” of information than each discrete “gain,” and conversely, that we are less likely to notice when we learn a lot at once than when we forget a lot at once, meaning that we could have very different distributions of informational (versus monetary) losses, with far fewer smaller values available for comparison. In fact, we might mainly attend to very large instances of forgetting, such as, for example, when we realize we forgot the entire plot of a book we recently read, making it more likely that any given loss of information we consider will seem small when compared to those large previous losses, suggesting that we could potentially see a reduction, if not a reversal, of loss aversion when it comes to information.

Finally, despite their intangibility, the nonmaterial outcomes examined in prior studies—e.g., time, environmental public goods, and romantic relationships—all carried obvious, and significant, value for the decision-makers involved. By contrast, the noninstrumental information used in our studies has no clear utility and thus cannot be considered a significant carrier of value. This distinguishing feature makes it very much an open, and fascinating, question whether loss aversion, the endowment effect, and gain-loss framing will be observed for noninstrumental information.

## Present Research

In three studies, we demonstrate that people treat gains and losses of (noninstrumental) information as they do gains and losses of goods by showing that preferences for information exhibit two fundamental features of Prospect Theory: loss aversion and the gain-loss framing effect. Studying information loss presents an empirical challenge. Although a sense of psychological ownership can develop for objects that are both material and nonmaterial in nature (50), evoking a sense of “loss” is more difficult for information than for goods since, once received and assimilated, information cannot be easily taken away. One possible analogy between information and physical goods concerns *realized* gains and losses, and equates owning (obtaining) a good with knowing (learning) information and losing a good with having information deleted from (long-term) memory; however, it is infeasible to selectively erase information in this way and therefore practically impossible to study experimentally. An alternative, more implementable analogy concerns *prospective* gains and losses—which are widely studied in decision-making research [e.g., (11, 16, 21, 22, 25, 26, 38, 39)]— and equates the prospect of gaining or losing a good of uncertain value (e.g., getting to play a gamble that offers a 50/50 chance to win $100 or $0) with the prospect of gaining or losing an unrevealed piece of information (e.g., getting to learn which country’s national animal is a unicorn). We exploit this latter analogy and create a sense of information loss by endowing participants with the expectation of learning facts—e.g., which US state forbids using other people’s Netflix accounts (see *SI Appendix* for the full set of facts used)—and confronting them with the prospect of losing that opportunity. The unit of information—analogous to a tangible good—is thus a single, unique fact. We simulate the experience of “owning” such a fact without actually revealing the information (i.e., the name of the state in the Netflix example) by setting the expectation that the missing information will be revealed. We then test whether (the prospect of) not learning the missing information one had expected to receive is given greater weight than (the prospect of) learning that same information with no prior expectation of receiving it, as predicted by loss aversion (51–53). Similarly, we test whether there is greater willingness to take risks to avoid not learning information one had expected to receive than to learn information one had not expected to receive, as predicted by diminishing sensitivity over gains and losses (16, 21).

We used interesting but essentially useless trivia-style facts in order to avoid some of the limitations inherent in prior research. Because the participants have no stake in the information and cannot translate it into any material rewards, we ensure that the observed preferences in our studies are for the information per se and not for anything else. These studies, therefore, provide robust evidence that loss aversion, the endowment effect, and the gain-loss framing effect also apply to information—not as a means to some reward, but as an end.

## Results

### Study 1.

**Fig. 1.**
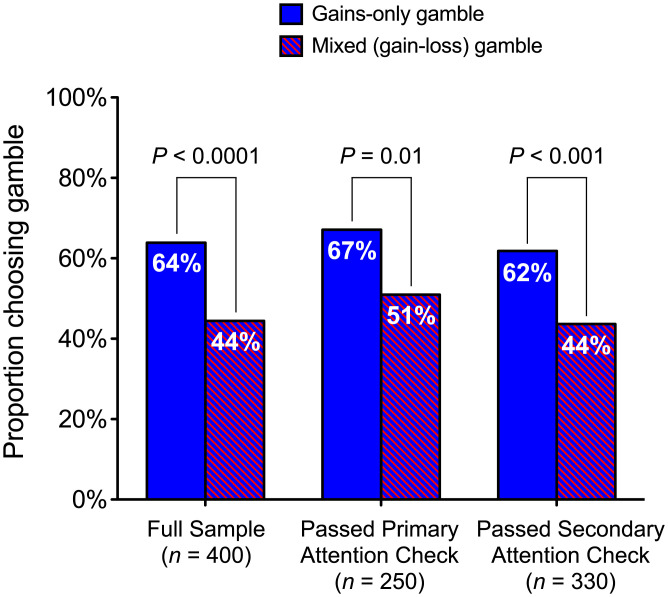
Study 1: the proportion of participants in each condition who chose the gamble, for the full sample and for two subsamples.

To determine whether preferences for information exhibit loss aversion (i.e., the disutility of losing information exceeds the utility of gaining it), the first (preregistered^‡^) experiment tested whether people are less likely to choose the same gamble when its two possible outcomes are framed as being a small gain and a small loss of information (mixed-frame), compared to when these are framed, respectively, as being a large gain and no gain of information (gains-only frame). All participants first saw six unrevealed facts, before being randomly assigned to one of two framing conditions. Participants in the gains-only frame condition were then given the choice to either (i) learn three of the facts for sure (the sure option) or (ii) instead opt for a 50/50 chance of learning all six facts or learning no facts (the gamble option). Participants in the mixed-frame condition were initially told that they were on course to learn three of the facts for sure and then given the choice to either (i) learn “their” three facts for sure (the sure option) or (ii) instead opt for a 50/50 chance of losing “their” three facts or learning three extra facts—the same likelihoods and final outcomes as the gamble option in the gains-only frame condition, but presented as offering either a loss or a gain of three facts relative to a reference point of three initial facts. Participants were less likely to choose the gamble in the mixed-frame condition (44%) than in the gains-only frame condition (64%) (χ^2^(1, *n* = 400) = 15.19, *P* < 0.0001, Φ = 0.195), demonstrating that the prospect of losing information is more aversive than the prospect of not gaining it (Fig. 1).

### Study 2.

The second experiment tested whether people exhibit the endowment effect for information. Participants were first presented with seven unrevealed trivia facts, which we randomly divided into two bundles of three and four facts. They were randomly assigned to one of two conditions: endowed or nonendowed. Participants in the nonendowed condition were simply offered a choice between the three- and four-fact bundle. Participants in the endowed condition, however, were initially told that they were on course to learn the three-fact bundle and then offered the option to learn the four-fact bundle instead. Thus, all participants were essentially given the choice to ultimately learn either the three-fact bundle or four-fact bundle, but half of them were endowed with the former.

**Fig. 2.**
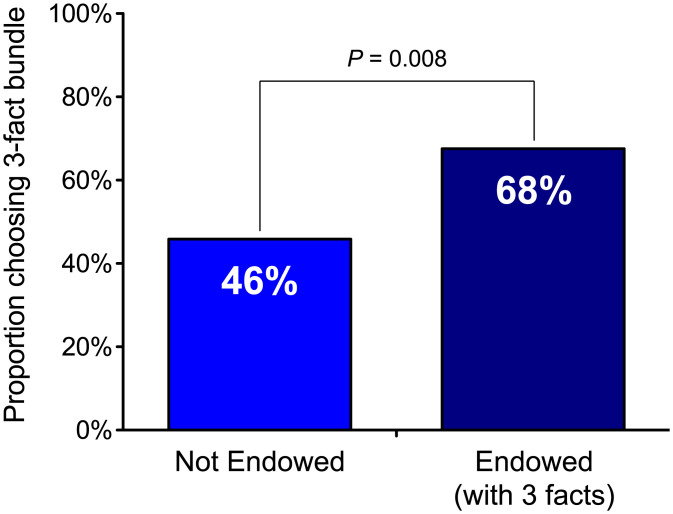
Study 2: the proportion of participants in each condition who chose the three-fact bundle.

While a minority of participants could rationally prefer the three-fact bundle when it happens to contain the fact(s) they are more curious about, randomizing facts across the two bundles for each participant ensured that most of them should rationally prefer the larger (four-fact) bundle over the smaller (three-fact) one. However, we predicted that participants endowed with the three-fact bundle would be less likely than nonendowed participants to choose the four-fact bundle, because doing so would feel to them like a loss—rather than foregone gain—of three facts. Indeed, participants in the endowed condition were more likely to prefer learning just three facts (68%) than those in the nonendowed condition (46%) (χ^2^(1, *n* = 146) = 7.03, *P* = 0.008, Φ = 0.219) (Fig. 2).

### Study 3.

The third (preregistered^§^) experiment tested whether information conforms to another property of Prospect Theory: differing risk preferences for uncertain outcomes framed as potential losses versus potential gains, relative to a reference point. To the extent that people exhibit this gain-loss framing effect for information, they should be more risk seeking when faced with potential losses of information than with equivalent potential gains. Participants were presented with a set of three unrevealed facts—either about US state laws, national customs, or foreign languages—before being randomly assigned to one of two framing conditions. Participants in the gain-frame condition chose between either (i) learning one fact for sure (smaller certain gain) or (ii) a gamble offering a one-third chance of learning three facts and a two-thirds chance of learning no facts (larger uncertain gain). Participants in the loss-frame condition chose between either (i) losing two facts for sure (smaller certain loss) or (ii) a gamble offering a one-third chance of losing no facts and a two-thirds chance of losing all three facts (larger uncertain loss).

**Fig. 3.**
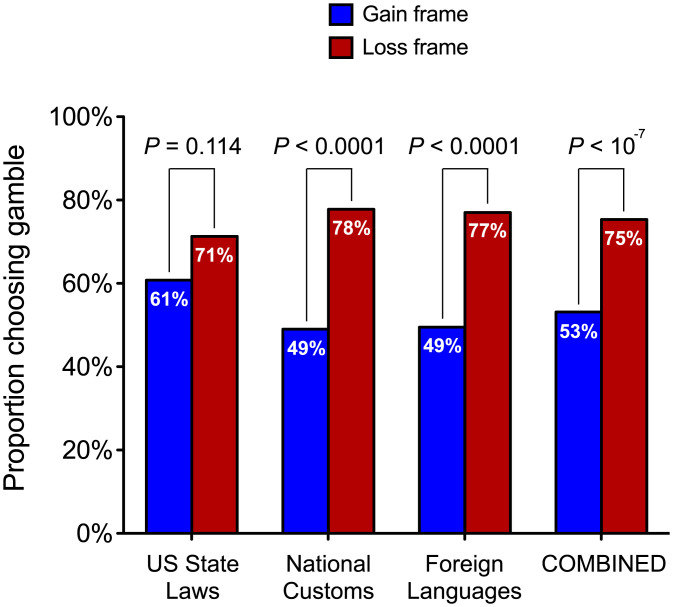
Study 3: the proportion of participants in each condition who chose the gamble, for each information topic and for all three topics combined.

Overall, participants were more likely to choose the gamble in the loss-frame than in the gain-frame condition (χ^2^(1, *n* = 601) = 32.16, *P* < 10^−7^, Φ = 0.231). Moreover, this tendency was observed for all three information topics and was significant for two of them: state laws: χ^2^(1, *n* = 203) = 2.50, *P* = 0.114, Φ = 0.111; customs: χ^2^(1, *n* = 201) = 17.86, *P* < 0.0001, Φ = 0.298; and languages: χ^2^ (1, *n* = 197) = 16.07, *P* < 0.0001, Φ = 0.286 (Fig. 3). We found the same gain-loss framing effect for two information topics (customs and languages) that has been observed for other outcomes, such as human lives (21): not only were participants less risk seeking when the information outcomes were framed as gains (versus losses), but they were actually slightly risk averse in the gain-frame condition, with just under 50% of participants choosing the risky option.^¶^

We conducted a logistic regression to test the effect of framing condition (dummy-coded: loss-frame = 1; gain-frame = 0) on risk preferences while controlling for information topic (with dummy variables for state laws and national customs, while setting foreign languages as the baseline), as well as basic demographic characteristics: age, gender, and education (dummy-coded: four-year college degree or higher = 1; less than a four-year college degree = 0). The loss-frame increased the likelihood of choosing the gamble (Exp(β) = 2.77, *P* < 0.001). We also found, consistent with prior studies (56), that men were significantly more likely to choose the gamble (Exp(β) = 1.47, *P* = 0.031), although this gender effect was smaller than our framing effect. There were no other significant main effects. (See SI Tables S1-S6 for regression analyses with demographic variables as controls for all three studies).

## Discussion

Economists have traditionally treated the value of information as derivative of its consequences for decision-making. While prior research on noninstrumental information has shown that this narrow view of information may be incomplete (7, 8, 11, 12), only a few accounts (57) have attempted to explain intrinsic preferences for information. One such account (58) argues that people seek (or avoid) information inasmuch as doing so helps them maintain their cherished beliefs. Another (59) proposes that people choose which information to seek or avoid by considering how it will impact their actions, affect, and cognition. Yet, outside of the curiosity literature (60), no existing account of information valuation considers preferences for information that has neither instrumental nor (concrete) hedonic value. By showing that key features of Prospect Theory’s value function also apply to individuals’ valuation of (even noninstrumental) information, the current paper suggests that we may also value information in some of the same fundamental ways that we value physical goods.

In our studies, we manipulate expectations of ownership rather than actual ownership; however, this is not necessarily a limitation since much of what we know about people’s typical preferences (including Prospect Theory) is derived from studies that used unresolved (and often hypothetical) gambles as the choice options, rather than actually endowing and taking away objects or financial payoffs. In fact, there is evidence that physical possession is not a prerequisite even for the endowment effect (61) and that psychological ownership—which can be triggered by anticipatory possession—may actually be responsible for loss aversion (62).

Nevertheless, future research could try to create a sense of information loss in other ways, perhaps by proving an assumed fact to be false or utilizing a memory interference task to facilitate the forgetting of recently learned information. Future research should also examine how we may be able to mitigate loss aversion for information, in an effort to encourage people to abandon inaccurate beliefs. For example, emotions such as sadness and disgust were found to negate or even reverse the endowment effect for objects (63), so it would be worthwhile to see if they do so for information as well.

We also leave to future research the questions of whether other preference patterns established for money and goods [e.g., ambiguity aversion (64, 65)] likewise apply to information and whether we can leverage the analogy with money and goods to determine the types of information people value more and most want to possess [e.g., information that is scarce or privileged (66)]. Furthermore, while the current studies focus on noninstrumental information, the preference patterns we observe likely also apply to instrumental information, in which case, our findings would have implications for a wide range of situations in which we want to encourage people to value useful information (e.g., many education and health care contexts). Our findings may also help guide research and shape policy on a critical modern issue, online consumer privacy, since the extent to which people can feel ownership over the personal data that firms collect (34) likely shapes their preferences for how such data are treated. In fact, identifying loss aversion and the endowment effect for information may be particularly relevant in the digital age, when unprecedented access to information complicates and potentially changes the way we value it—e.g., being able to easily look up a fact makes it less important to remember (67). But that makes the finding that we do indeed feel ownership over—and an accompanying loss aversion for—information all the more unexpected and interesting.

## Materials and Methods

Each of the three studies reported in this paper received ethical approval from the Carnegie Mellon University institutional review board. All participants provided informed consent before beginning the study. The datasets and survey materials for these studies are available on OSF at: https://osf.io/9smht/?view_only=8555702974fa4587ae2142174ba1b755.

In all three studies, participants were randomly assigned to one of two key conditions (detailed below, for each study) and presented with a choice between two options whose outcome(s) involved learning (or not learning) one or more unrevealed facts. Every participant’s choice was implemented for real, with any corresponding fact(s) being revealed to the person before the end of the study.

### Study 1.

We recruited 400 adult participants from Prolific, a crowdsourcing platform for scientific research (44% female, 55% male, 1% other; age: Range: 18-71, Median = 30, M = 32.3, SD = 11.0). Participants saw six unrevealed facts and were randomly assigned to either a mixed-frame or a gains-only frame condition, which differed in two aspects. First, in the mixed-frame condition, we created the expectation of information ownership: participants were told they were on course to learn three of the six facts, selected at random, and they waited 5 s to learn which facts had been selected. This step was designed to create a sense of ownership over those three facts, from which we could engender a sense of loss. Participants in the gains-only frame, by contrast, were not initially endowed with the prospect of having three facts revealed.

Second, all participants were given the option to learn three facts for certain or to instead take a gamble that offered an equal (50/50) chance to end up learning either (i) all six facts or (ii) zero facts. However, in the mixed-frame condition, the former possible gamble outcome was described as a gain of three extra facts (in addition to the three they were already on course to learn), while the latter possible gamble outcome was described as a loss of the three facts participants were on course to learn. In the gains-only frame condition, those same two possible gamble outcomes were instead described as a gain of six facts and zero facts, respectively (Table 1).

**Table 1.** Study 1: Choice options presented in each condition

Mixed-frame	Gains-only frame
A: Get your three facts for sure. B: Toss a coin: Heads: Get three additional facts. Tails: Lose your three facts (get no facts).	A: Get these three facts for sure. B: Toss a coin: Heads: Get six facts. Tails: Get no facts.

In both conditions, the three unrevealed facts that participants would learn for sure if they rejected the gamble were randomly selected from the full list of six unrevealed facts, and all participants also saw the other three unrevealed facts they would learn if they chose the gamble and won. Their two choice options were therefore objectively equivalent in both conditions, with the only difference being that our endowment and framing manipulations made participants in the mixed-frame condition feel that they faced a potential loss if they chose the gamble.

Immediately after making their choice, participants completed an attention/comprehension check to determine whether they understood the choice they had faced. Our results replicate if we only include those who passed this check (*n_pass_* = 250). Our study also contained a secondary, unrelated attention check question, and our results again replicate if we only include those who passed this other question (*n_pass_* = 330) (Fig. 1; see SI Table S7 for chi-square results for both sub-samples).

### Study 2.

We recruited 146 adult participants from Prolific (45% female, 54% male, 1% other; age: Range: 18-74, Median = 29, M = 33.0, SD = 12.6) and randomly assigned them to one of two conditions: endowed or nonendowed. We showed participants seven unrevealed facts and gave them the choice to learn a randomly selected subset of either three or four of these facts. After we presented the seven unrevealed facts, but before we divided them into the two subset bundles, participants in the endowed condition were initially told that they were on course to learn three of the facts, selected at random. As in Study 1, this was designed to create a feeling of ownership over the facts without participants actually learning the information. Participants in the nonendowed condition skipped this endowment phase. Every participant then chose which bundle of facts (three or four) they wanted revealed.

### Study 3.

We randomly assigned 601 adult participants from Prolific (49% female, 51% male, 1% other; age: Range: 18-77, Median = 33, M = 36.3, SD = 12.6) to see one of three sets of unrevealed facts about either (i) US state laws, (ii) national customs, or (iii) foreign languages. We gave all participants the choice between (i) having one of the three facts revealed for sure (smaller certain outcome) or (ii) a gamble offering a one-third chance of having all three facts revealed and a two-thirds chance of having no facts revealed (larger uncertain outcome).

We modeled our experiment on the classic “Asian Disease” problem, which Tversky and Kahneman (21) [see also (22)] originally used to demonstrate the gain-loss framing effect. Participants were randomly assigned to either a gain- or a loss-frame condition, resulting in a 2 (framing condition) × 3 (information topic) fully-between-subjects experimental design. In the gain-frame condition, we described the choice outcomes in terms of the number of facts they could learn, whereas in the loss-frame condition, we described their choice outcomes in terms of the number of facts they would *not* learn. Specifically, participants in the gain-frame were told that if they chose the sure option, we would reveal one fact for certain, whereas if they chose the gamble, there was a one-third chance that we would reveal all three facts and a two-thirds chance that we would not reveal any of the facts. Participants in the loss-frame were instead told that if they chose the sure option, two of the three facts would be blacked out (i.e., permanently hidden) for certain, whereas if they chose the gamble, there was a one-third chance that none of the missing information would be blacked out and a two-thirds chance that all of it would be blacked out.

We showed participants an example of what they would see if they chose the sure option (Table 2). As an illustration, we randomly selected one of the three unrevealed facts as the one they might learn (and represented the missing information with the text: “Answer here”). For the two remaining unrevealed facts, we represented the missing information in one of two ways: In the gain-frame condition, a blank space underlined in red was placed where the missing information would have appeared, suggesting that the missing information had not yet been populated (i.e., gained). In the loss-frame condition, the missing information was covered by a thick black line, as in a redacted document, suggesting that the missing information had been populated but was then permanently obscured (i.e., lost).

**Table 2.** Study 3: The example shown to participants in each framing condition, illustrating what they would see if they chose to have one of the facts revealed with certainty

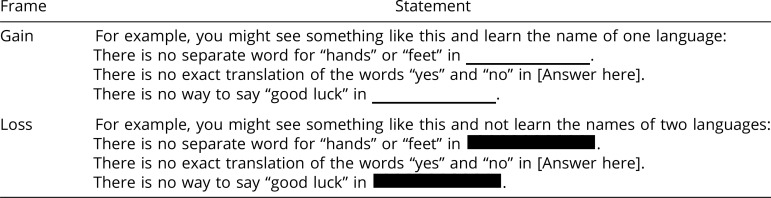

After making their choice, but before learning the outcome(s), participants answered an attention/comprehension check designed to evaluate whether they understood both choice options. In both framing conditions, only 5% of participants failed this check, and excluding them did not alter the significance of the results (see SI Table S8 for these results).

We conducted a follow-up experiment (see Supporting Information) to test whether redacting (i.e., visually occluding) the missing information in the loss-frame condition might be increasing curiosity for the information—a possible alternative explanation for our findings. Participants were presented with three unrevealed facts about US state laws in one of the two representation formats used in Study 3—either the missing fact format from the gain-frame condition or the blacked-out fact format from the loss-frame condition—, and they (i) rated how curious they were about the three facts and (ii) were offered the opportunity to complete a simple effort task in order to reveal all three facts. We found that participants were neither more curious nor more willing to complete the effort task in the blacked-out fact format; if anything, they were (directionally) more curious and (directionally) more willing to complete the effort task in the missing fact format. Thus, the findings of Study 3 are not attributable to a greater curiosity for occluded information.

## Supplementary Material

Supplementary File

## Data Availability

Data files and study materials have been deposited in OSF (https://osf.io/9smht/?view_only=8555702974fa4587ae2142174ba1b755) (68).

## Footnotes

^*^Specifically, they conclude their paper by writing, “After more than a decade of research on this topic we have become convinced that the endowment effect, status quo bias, and the aversion to losses are both robust and important. Then again, we admit that the idea is now part of our endowment, and we are naturally keener to retain it than others might be to acquire it.” (p. 205)

^†^It is worth noting that a growing body of research has challenged the notion that these properties of Prospect Theory are ubiquitous, by demonstrating important boundary conditions for loss aversion (40–42), the endowment effect (35, 43, 44), and the gain-loss framing effect (25, 45, 46).

^‡^Study IDs on AsPredicted.com: Study 1 = 42280

^§^Study IDs on AsPredicted.com: Study 3 = 34314

^¶^Given that our study involves small numbers of outcomes (one to three facts), finding that only a slight majority of participants were risk averse in the gain-frame condition is consistent with prior work showing that people become more risk seeking, across both frames, with smaller numbers of outcomes (e.g., six lives at stake instead of 600) (54, 55). Moreover, an additional possible explanation for why the majority of participants chose the gamble in both the gain- and loss-frame conditions for US state laws is that—for our US-based participants—the value of the full set of these facts may seem greater than the sum of its parts because it allows for state-to-state comparisons and/or increases the chance of learning something interesting about one’s own state.
