# Supplementary material for: Loss aversion, the endowment effect, and gain-loss framing shape preferences for noninstrumental information
Source: Proc Natl Acad Sci U S A. 2022 Aug 16;119(34):e2202700119. doi: 10.1073/pnas.2202700119 (PMC9407664; doi:10.1073/pnas.2202700119)
Supplement: Supplementary File [file pnas.2202700119.sapp.pdf]

PNAS

Supporting Information for

Loss aversion, the endowment effect, and gain-loss framing shape preferences for non-instrumental information

Yana Litovsky*[1] George Loewenstein[2] Samantha Horn[2] Christopher Y. Olivola[2,3]

[1] Department of Banking and Finance, University of Innsbruck, Innsbruck, Austria, 6020

[2] Department of Social and Decision Sciences, Carnegie Mellon University, Pittsburgh, PA, 15213, USA

[3] Tepper School of Business, Carnegie Mellon University, Pittsburgh, PA, 15213, USA

* To whom correspondence may be addressed. Email: Yana.Litovsky@uibk.ac.at.

Contents

Appendix Table: Experimental stimuli Page 2

Tables S1-S6: Regression analyses for Studies 1-3 Pages 3-4

Tables S7-S8: Chi-square analyses for participants who passed attention checks Page 5

Curiosity Study (follow-up to Study 3): Methods and results Pages 6-7

Appendix: Experimental stimuli used in each study.

**Items used in Studies 1 & 2**	In this U.S. state you cannot use someone else's Netflix account. *[Answer: Tennessee]*	
	This animal uses flowers, glass and plastic to decorate its dwelling. *[Answer: Bowerbird]*	
	In this language there is no separate word for "hands" or "feet." *[Answer: Russian]*	
	This country recognizes pillow fighting as an official sport. *[Answer: Canada]*	
	This brutal dictator was nominated for the Nobel peace prize two times. *[Answer: Stalin]*	
	This country's national animal is a unicorn. *[Answer: Scotland]*	
	In this country it is traditional to eat KFC for Christmas.* *[Answer: Japan]*	
**Items used in Study 3**	US State Laws	It is illegal to dance to the national anthem in in this U.S. state. *[Answer: Massachusetts]*
		Anyone with a sexually transmitted disease cannot get married in in this U.S. state. *[Answer: Nebraska]*
		You cannot use someone else's Netflix account in in this U.S. state. *[Answer: Tennessee]*
	National Customs	It is considered bad luck to write someone's name in red ink in this country. *[Answer: Korea]*
		People point with their lips instead of their finger in this country. *[Answer: Nicaragua]*
		It is traditional to eat KFC for Christmas in this country. *[Answer: Japan]*
	Foreign Languages	There is no separate word for "hands" or "feet" in this language. *[Answer: Russian]*
		There is no exact translation of the words "yes" and "no" in this language. *[Answer: Irish]*
		There is no way to say "good luck" in this language. *[Answer: Japanese]*

** This item was only used in Study 2.*

Tables S1-S6
Logistic regressions (with robust standard errors) conducted as robustness checks for Studies 1-3, with demographic variables simultaneously entered as controls.

Table S1: Study 1 logistic regression results (*N* = 397)

**Dependent Variable:** Participant choice (1 = gamble; 0 = sure outcome)					
Predictors	*B*	*Exp(B)*	*SE*	*z-statistic*	*P-value*
Mixed (gain-loss) frame	-0.766	0.465	0.207	-3.700	< 0.001
Age	0.010	1.010	0.010	0.990	0.321
Male (vs. Female)	0.168	1.183	0.209	0.800	0.422
College degree*	-0.187	0.829	0.232	-0.810	0.420

* Baseline: Less than a 4-year undergraduate degree.

Table S2: Study 1 logistic regression results, excluding participants who failed the primary attention check (*N* = 247)

**Dependent Variable:** Participant choice (1 = gamble; 0 = sure outcome)					
Predictors	*B*	*Exp(B)*	*SE*	*z-statistic*	*P-value*
Mixed (gain-loss) frame	-0.656	0.519	0.271	-2.420	0.016
Age	0.023	1.023	0.013	1.760	0.079
Male (vs. Female)	0.070	1.073	0.271	0.260	0.796
College degree*	-0.347	0.707	0.295	-1.180	0.240

* Baseline: Less than a 4-year undergraduate degree.

Table S3: Study 1 logistic regression results, excluding participants who failed the secondary attention check (*N* = 327)

**Dependent Variable:** Participant choice (1 = gamble; 0 = sure outcome)					
Predictors	*B*	*Exp(B)*	*SE*	*z-statistic*	*P-value*
Mixed (gain-loss) frame	-0.700	0.496	0.228	-3.070	0.002
Age	0.018	1.018	0.011	1.610	0.108
Male (vs. Female)	0.186	1.204	0.228	0.820	0.415
College degree*	-0.108	0.897	0.250	-0.430	0.666

* Baseline: Less than a 4-year undergraduate degree.

Table S4: Study 2 logistic regression results (*N* = 145)

**Dependent Variable:** Participant choice (1 = 3-fact bundle; 0 = 4-fact bundle)					
Predictors	*B*	*Exp(B)*	*SE*	*z-statistic*	*P-value*
Endowed condition	0.921	2.512	0.358	2.570	0.010
Age	-0.006	0.994	0.014	-0.400	0.691
Male (vs. Female)	0.588	1.800	0.362	1.620	0.104
College degree*	-0.439	0.645	0.354	-1.240	0.216

* Baseline: Less than a 4-year undergraduate degree.

Table S5: Study 3 logistic regression results (*N* = 597)

**Dependent Variable:** Participant choice (1 = gamble; 0 = sure outcome)					
Predictors	*B*	*Exp(B)*	*SE*	*z-statistic*	*P-value*
Loss frame	1.018	2.768	0.181	5.620	< 0.001
Topic: US State Laws	0.152	1.164	0.219	0.690	0.488
Topic: National Customs	0.060	1.062	0.214	0.280	0.778
Age	-0.007	0.993	0.007	-0.950	0.342
Male (vs. Female)	0.386	1.471	0.178	2.160	0.031
College degree*	-0.027	0.974	0.180	-0.150	0.883

* Baseline: Less than a 4-year undergraduate degree.

Table S6: Study 3 logistic regression results, excluding participants who failed the attention check (*N* = 565)

**Dependent Variable:** Participant choice (1 = gamble; 0 = sure outcome)					
Predictors	*B*	*Exp(B)*	*SE*	*z-statistic*	*P-value*
Loss frame	1.094	2.987	0.190	5.760	< 0.001
Topic: US State Laws	0.174	1.190	0.229	0.760	0.447
Topic: National Customs	0.087	1.091	0.223	0.390	0.697
Age	-0.009	0.991	0.008	-1.150	0.249
Male (vs. Female)	0.432	1.541	0.186	2.320	0.020
College degree*	-0.012	0.988	0.188	-0.060	0.948

* Baseline: Less than a 4-year undergraduate degree.

Table S7: Study 1 choice proportions and chi-square results, for participants who passed the primary or secondary attention check

**Dependent Variable:** Participant choice (1 = gamble; 0 = sure outcome)					
Topic	*Gains-only gamble frame*	*Mixed (gain-loss) gamble frame*	χ^2	*N*	*P-value*
Attention check passed:					
Primary	**67%**	**51%**	6.63	250	0.010
Secondary	**62%**	**44%**	10.94	330	< 0.001

Table S8: Study 3 choice proportions and chi-square results, excluding participants who failed the attention check

**Dependent Variable:** Participant choice (1 = gamble; 0 = sure outcome)					
Topic	*Gain-frame*	*Loss-frame*	χ^2	*N*	*P-value*
US State Laws	**62%**	**74%**	3.10	189	0.078
National Customs	**50%**	**80%**	18.61	192	< 0.0001
Foreign Languages	**51%**	**78%**	15.33	188	< 0.0001
COMBINED	**54%**	**77%**	33.55	569	$< 10^{-8}$

Curiosity Study (follow-up to Study 3)

Methods:

We recruited 237 Carnegie Mellon University students (63% female, 36% male, 2% other; age: Range: 18-68, Median = 20, M = 23.4, SD = 9.7), mostly undergraduates, to participate in this study in exchange for course credit. All participants were shown three facts about unusual US State Laws (the same ones used in Study 3), but, as in Study 3, we did not reveal the names of those states (see Appendix, above, for the exact stimuli). Participants were randomly assigned to one of two conditions, which varied how the missing information (i.e., the state name) was represented. In the missing fact format condition, the missing information was represented by a blank space underlined in red where it would have appeared—the same format used in the gain-frame in Study 3. In the blacked-out fact format condition, the missing information was covered by a thick black line, as in a redacted document—the same format used in the loss-frame in Study 3.

Participants then rated how curious they were to learn these three facts (i.e., to learn the names of the states) on a 1-7 scale, ranging from "Not at all curious" to "Very curious." Finally, they were given the opportunity to learn all three facts by completing a simple effort task: moving five sliders to specified numbers ranging from 1 to 10. If a participant chose to skip a slider or moved it to an incorrect number, s/he finished the study without learning the three facts. If (and only if) a participant moved all five sliders correctly, s/he learned all three facts. Curiosity ratings were positively correlated with both the number of sliders completed ($r = 0.28$, $P < 0.0001$) and the likelihood of completing the entire effort task (i.e., all five sliders) ($r = 0.29$, $P < 10^{-5}$). In other words, a greater curiosity about the unrevealed facts predicted a greater willingness to exert effort to reveal those facts.

Results:

There were no significant differences, between formatting conditions, in either the curiosity ratings or the numbers of sliders completed (Fig. S1). Participants were directionally, but only marginally significantly, more curious to learn the three facts in the missing fact format condition (M = 5.40, SD = 1.62) than in the blacked-out fact format condition (M = 4.98, SD = 1.72) ($t(235) = 1.93$, $P = 0.055$). The number of sliders completed was also directionally, but not significantly, greater in the missing fact format condition (M = 4.82, SD = 0.84) than in the blacked-out fact format condition (M = 4.63, SD = 1.26) ($t(235) = 1.35$, $P = 0.177$). Similarly, participants were directionally, but not significantly, more likely to complete the entire effort task (i.e., move all five sliders to their indicated numbers) in the missing fact format condition (95%) than in the blacked-out fact format condition (92%) ($\chi^2(1, n = 237) = 0.772$, $P = 0.379$).

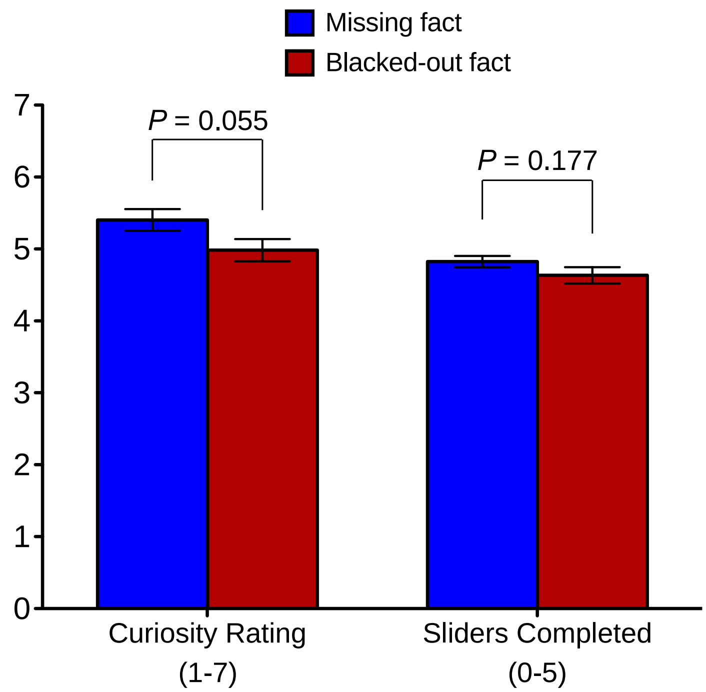

Fig. S1. Curiosity Study: Average curiosity rating (left bars) and average number of sliders completed (right bars), as a function of format.
